# Extremely fast and incredibly close: cotranscriptional splicing in budding yeast

**DOI:** 10.1261/rna.060830.117

**Published:** 2017-05

**Authors:** Edward W.J. Wallace, Jean D. Beggs

**Affiliations:** 1 School of Informatics, University of Edinburgh, EH8 9AB, United Kingdom; 2 Wellcome Trust Centre for Cell Biology, University of Edinburgh, EH9 3BF, United Kingdom

**Keywords:** CTD phosphorylation, introns, polymerase CTD, polymerase pausing, splicing kinetics

## Abstract

RNA splicing, an essential part of eukaryotic pre-messenger RNA processing, can be simultaneous with transcription by RNA polymerase II. Here, we compare and review independent next-generation sequencing methods that jointly quantify transcription and splicing in budding yeast. For many yeast transcripts, splicing is fast, taking place within seconds of intron transcription, while polymerase is within a few dozens of nucleotides of the 3′ splice site. Ribosomal protein transcripts are spliced particularly fast and cotranscriptionally. However, some transcripts are spliced inefficiently or mainly post-transcriptionally. Intron-mediated regulation of some genes is likely to be cotranscriptional. We suggest that intermediates of the splicing reaction, missing from current data sets, may hold key information about splicing kinetics.

## INTRODUCTION

RNA splicing is an essential process in the maturation of most transcripts produced by eukaryotic RNA polymerase II (Pol II). RNA molecules can be spliced while still being transcribed, as shown by pioneering electron micrography studies of *Drosophila* embryo transcripts (Beyer and Osheim 1988; Beyer et al. 1981). Since then, diverse experimental methods have provided extensive support for functional coupling between splicing and transcription (for review, see Alexander and Beggs 2010; Naftelberg et al. 2015; Alpert et al. 2016; Saldi et al. 2016). Evidently, transcription can affect splicing and vice versa, but how this is achieved and regulated is largely unknown. The speed of splicing varies from gene to gene, depending on the strength of splice sites as well as other factors, and expression of some genes is regulated through splicing. What are the transcriptome-wide patterns of splicing kinetics?

Recently, distinct next-generation sequencing approaches have measured the coupling of transcription to splicing in *Saccharomyces cerevisiae*: fast metabolic labeling with 4-thio-uracil (4tU-seq) (Barrass et al. 2015), nascent RNA-seq (Harlen et al. 2016), and single molecule intron tracking (SMIT) (Carrillo Oesterreich et al. 2016). These approaches produce, for many genes, quantitative estimates of the speed of splicing, extent of cotranscriptional splicing, or polymerase position at splicing, respectively. Collectively, these data demonstrate that, in budding yeast, many introns are spliced out very soon after the intron is transcribed, a scenario that was controversial 10 years ago. Alternative next-generation sequencing methods measure polymerase position, without so far providing high-resolution measures of splicing: nascent elongating transcript sequencing (NET-seq) (Harlen et al. 2016), and crosslinking to modified Pol II and analysis of cDNA (mCRAC) (Milligan et al. 2016). Importantly, Harlen and colleagues and Milligan and colleagues also measure how the phosphorylation state of the carboxy-terminal domain (CTD) of a Pol II large subunit correlates with splicing factor recruitment or intron position genome-wide.

Here, we discuss the strengths and limitations of each approach, and the extent to which their results agree. One remarkable point of agreement is that ribosomal protein transcripts, the largest and most abundant class of spliced mRNAs, tend to be spliced faster and more cotranscriptionally.

## METHODS OF MEASURING NASCENT RNA

### 4tU-seq (Barrass et al. 2015)

4tU-seq uses short-timescale metabolic labeling to measure the first minutes of RNA transcription and processing. Nascent transcripts are labeled by incorporation of the uracil analog 4tU. After flash-freezing and cell lysis, the 4tU-labeled transcripts are biotinylated and then isolated based on their affinity for streptavidin beads, followed by randomly primed reverse transcription and sequencing. Using a statistical model to compare the spliced:unspliced ratio of transcripts labeled with 4tU for different times, the “relative speed of splicing” of each transcript is determined. The reported measure is the area under the curve (AUC) of the spliced:unspliced ratio, a time-weighted average of the fraction spliced within 5 min of synthesis. Note that the overall rate of conversion of pre-mRNA to spliced mRNA is determined in real time rather than relative to the movement of Pol II.

As 4tU-seq is essentially RNA-seq applied to the subset of RNA that is newly synthesized, a range of mature, validated tools are available to analyze the data, including building on the MISO algorithm for mRNA isoform quantification (Katz et al. 2010), to accommodate time-series labeling (DICE) (Huang and Sanguinetti 2016). These statistical tools provide robust gene-level quantification of splicing by combining strong information from the small proportion of reads that span a splice junction with individually weak information from the majority of nonjunction reads. However, 4tU-seq is uninformative about splicing intermediates (the products of the first catalytic step) or excised introns (the by-product of the second catalytic step): Indeed, at these short timescales the intron:exon ratio may detect variability in degradation rates of excised introns as well as variability in splicing rates (Box 1).

**BOX 1. Difficulties in detecting all stages of splicing**

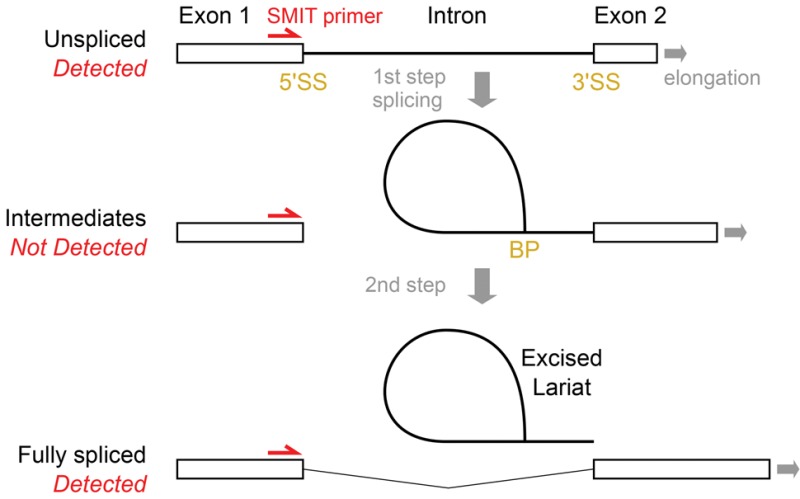
The pre-mRNA splicing reaction has two catalytic steps: In the first step, the 5′SS is cleaved and the lariat intron–exon intermediate species is formed by joining at the branch point (BP); and in the second, the 3′ end of the upstream exon is joined to the downstream exon at the 3′SS, resulting in spliced mRNA and an excised lariat intron. Biased detection of these various RNA species complicates analysis in all methods discussed here.

4tU-seq quantifies splicing by the ratio of exonic to all reads for a given RNA, as well as incorporating the unique information from junction reads, implicitly assuming that excised introns are degraded quickly enough that most intronic reads are from unspliced pre-mRNA. Although excised introns are, in general, quickly degraded, some lariats might be degraded on a timescale comparable to that of 4tU-seq measurements. Indeed, some evolutionarily conserved noncoding RNAs, including snoRNAs, are processed from introns, and these may be degraded slowly (Hooks et al. 2016). Therefore, 4tU-seq may *underestimate* the rate of splicing for transcripts with stable intron-derived products.

SMIT quantifies splicing by sequencing PCR products that extend from a primer site upstream of the 5′SS to the 3′ Pol II position. For splicing intermediates, these sites are not contiguous, and so are not detectable by SMIT. Therefore, SMIT may *overestimate* the extent of splicing of transcripts for which lariat intermediates represent a significant fraction of the total (slow second step), and overestimate the speed of splicing if spliceosome assembly and the first step of splicing occur before the 3′SS is transcribed (first step splicing is possible once the BP becomes available), such that distance from the 3′SS does not reflect all aspects of splicing.

Consider nascent transcripts whose 3′ end is at position *n*. If the true count of pre-mRNA is *P*_*n*_, splicing intermediates is *I*_*n*_, and fully spliced mRNA is *F*_*n*_, then the fraction that has completed splicing is *F*_*n*_/(*P*_*n*_ + *I*_*n*_ + *F*_*n*_). However, SMIT estimates the fraction spliced as *F*_*n*_/(*P*_*n*_ + *F*_*n*_), which is strictly greater; the difference depends on *I*_*n*_.

This may explain a puzzle in the SMIT analysis, that some estimates of the position of onset of splicing conflict with structural models. The *average* position for onset (10% of transcripts spliced) of 26 nt after transcription of the 3′ splice site is remarkably soon given that the distance between polymerase and spliceosome active sites is estimated as 24 nt (Carrillo Oesterreich et al. 2016). Perhaps this is not surprising: In a first-order kinetics approximation of splicing, the relative time or position of splicing would be an exponential distribution, with a mode at the first available point. However, beyond averages, SMIT's quantification algorithm reports that 24 of 87 measured genes have splicing onset <24 nt after the 3′ SS, 9 are 50% spliced by then, and onset is not reported for a further 31 genes.

NET-seq detects Pol II-associated 3′ ends of RNA species, including many reads exactly at the 3′SS which are most likely from excised introns attached to spliceosomes that are still associated with Pol II (Harlen et al. 2016). Interestingly, an earlier NET-seq data set observed an abundance of 3′ end reads exactly at the 5′SS (Churchman and Weissman 2011), also consistent with a population of splicing intermediates that pull down with elongating Pol II.

Complementary methods quantify splicing intermediates by sequencing, even in wild-type cells (Gould et al. 2016; Qin et al. 2016). However, these rely on enrichment of lariats, so provide weak information on the kinetics of splicing. Methods that measure all steps of the cotranscriptional splicing reaction simultaneously are thus needed to fill in the gaps in our quantitative understanding of cotranscriptional splicing.

### SMIT (Carrillo Oesterreich et al. 2016)

The SMIT approach cleverly exploits paired-end RNA sequencing to measure the splicing status of nascent transcripts that are assumed to be still associated with Pol II. Cells are harvested by centrifugation and washed in cold buffer, requiring the assumption that transcription and splicing are arrested to the same degree during sample preparation, then chromatin is isolated in a multistep procedure developed earlier by the same group (Carrillo Oesterreich et al. 2010), and mature poly(A)^+^ RNA is further depleted. Remaining chromatin-derived RNA is reverse-transcribed from a 3′ end-ligated oligonucleotide, then PCR amplified, using gene-specific primers upstream of target introns to ensure high read-depth for target transcripts. Most budding yeast intron-containing genes have short first exons, therefore 87 target genes were selected based on the suitability of first exons to prime PCR across the intron. To aid correct quantification despite dense sampling of target genes, random molecular barcodes were incorporated in the ligated oligonucleotide. By design, the 3′ end reads report polymerase position, while the 5′ end reads, because they start close to the introns, report splicing status. SMIT detects intron-containing transcripts prior to the first step of splicing and also detects spliced transcripts that have completed the second step of splicing (exon joining, Box 1). SMIT cannot detect transcripts that have undergone only the first step of splicing, for which the 3′ end of nascent RNA is not contiguous with the 5′ primer site (Box 1). The fraction of transcripts that have undergone both steps is calculated at spatially binned Pol II positions for each tested gene, and the position is estimated at which 10%, 50%, and 90% of nascent transcripts are spliced comparably to “saturation,” i.e., fraction spliced for the most 3′ Pol II positions. Thus SMIT reports the “distribution of Pol II positions at splicing.” SMIT's measurements of relative positions of polymerase and splicing are analogous to those made from the electron micrograph tracings of Beyer and Osheim (1988) and Beyer et al. (1981).

Importantly, Carrillo Oesterreich et al. (2010) validate the SMIT assay by comparison to other methods: Long-read sequencing confirms the onset of splicing soon after polymerase passes the 3′SS. Further, SMIT shows that splicing moves further 3′ from the 3′SS in a fast-elongating Pol II mutant, as predicted by kinetic competition of polymerization and splicing.

### Nascent RNA-seq (Harlen et al. 2016)

For nascent RNA-seq, cells with affinity-tagged Pol II are flash-frozen and lysed, Pol II is immunoprecipitated, and the associated, nascent RNA transcripts are purified, fragmented, and sequenced. This approach allows estimation of the fraction of RNA spliced cotranscriptionally, but does not provide information about Pol II occupancy or splicing kinetics. Here we compare three estimates of the fraction spliced from the same data: from the fraction of intronic reads within 25 nt of the 3′SS (“3′SS int:ex”), from reads spanning the splice junction, or recalculated using all reads by DICE (Huang and Sanguinetti 2016). Only a small proportion of nascent RNA-seq reads span a splice junction or align within 25 nt of the 3′SS, thus for less abundant transcripts the fraction spliced cotranscriptionally is the ratio of two small numbers that are susceptible to count noise. More recent, noise-aware methods such as DICE circumvent this issue by pooling information from junction, intron, and exon reads.

### NET-seq (Churchman and Weissman 2011; Harlen et al. 2016)

NET-seq quantifies Pol II occupancy genome-wide by sequencing the 3′ ends of all transcripts associated with Pol II (Churchman and Weissman 2011). Similar to nascent RNA-seq, RNA is extracted from immunoprecipitated Pol II, the crucial difference being that short reads (75 nt) are obtained from the 3′ ends of extracted RNA that has not been fragmented. As only a very small fraction of all nascent transcripts are spliced within these short distances, there is a very small number of spliced reads, so this approach is not suited to measuring splicing relative to the Pol II position.

Detection of tRNA, which is produced by RNA Pol III, and accumulation of reads at the 3′ ends of snoRNAs, suggests detectable levels of contamination from mature transcripts in NET-seq data. Similarly, 4tU-seq detects non-Pol II transcripts by design, and low levels of background RNA are isolated even without 4tU-labeling.

### mCRAC (Milligan et al. 2016)

With CRAC (cross-linking and analysis of cDNA) (Granneman et al. 2009), short fragments of RNA are identified that are UV crosslinked to, and protected from nuclease digestion by, a tagged bait protein. The protocol is analogous to CLIP (Darnell 2010), but features a stringent denaturation step to reduce background. Modification CRAC (mCRAC) uses a two-step purification, first with tagged Pol II, then with antibodies specific to different phosphorylation states of Pol II's CTD, to obtain fragments of nascent transcripts associated with distinct states of the elongation machinery. Importantly, these data were used as input to a Bayesian model that suggests multiple distinct initiation and elongation states of Pol II. We address this data set only briefly here on account of its relatively low depth of sequencing that does not permit an assessment of cotranscriptional splicing.

The data obtained by these approaches measure many aspects of transcription beyond those coupled to splicing: 4tU-seq, NET-seq, and mCRAC allow estimates of transcription rates as distinct from RNA abundance, including antisense and unstable transcripts, and a comparison would be instructive. However, we focus here on coupling of transcription and splicing.

## RESULTS

### Ribosomal protein genes tend to be spliced fast and cotranscriptionally

**FIGURE 1.**
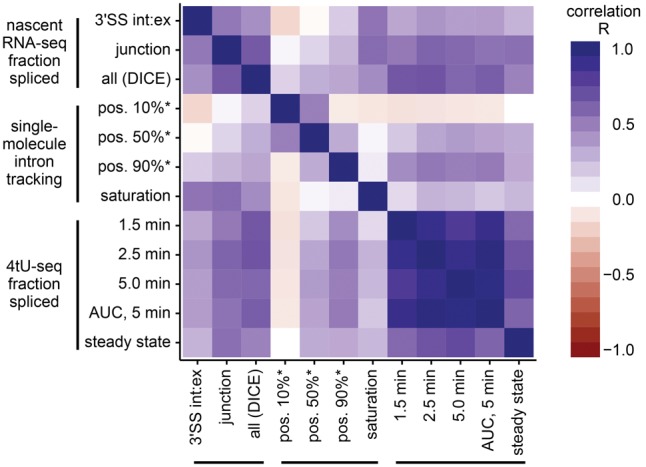
Estimates of cotranscriptional splicing, or splicing speed, mostly agree. Methods of measuring nascent RNA are described in the text; here we plot Pearson's correlation between genewise measurements. As expected, higher fraction cotranscriptionally spliced corresponds to smaller position of splicing, so for visual clarity, we reversed the sign of correlations for rows and columns containing position-based measurements (*).

There is a great deal of agreement between results of these distinct methods (Fig. 1). As with most high-throughput data, between-study correlations are generally lower than within-study correlations. Using DICE to estimate the nascent RNA-seq fraction that is spliced generally correlates better with other studies; however, the nascent RNA-seq estimates that focus on the 3′SS reads correlate better with SMIT's saturation values, which also focus on the 3′ end of the gene. SMIT's positions for near-complete (90%) splicing correlate better with alternative measurements than the positions of onset (10%) or median (50%) splicing.

**FIGURE 2.**
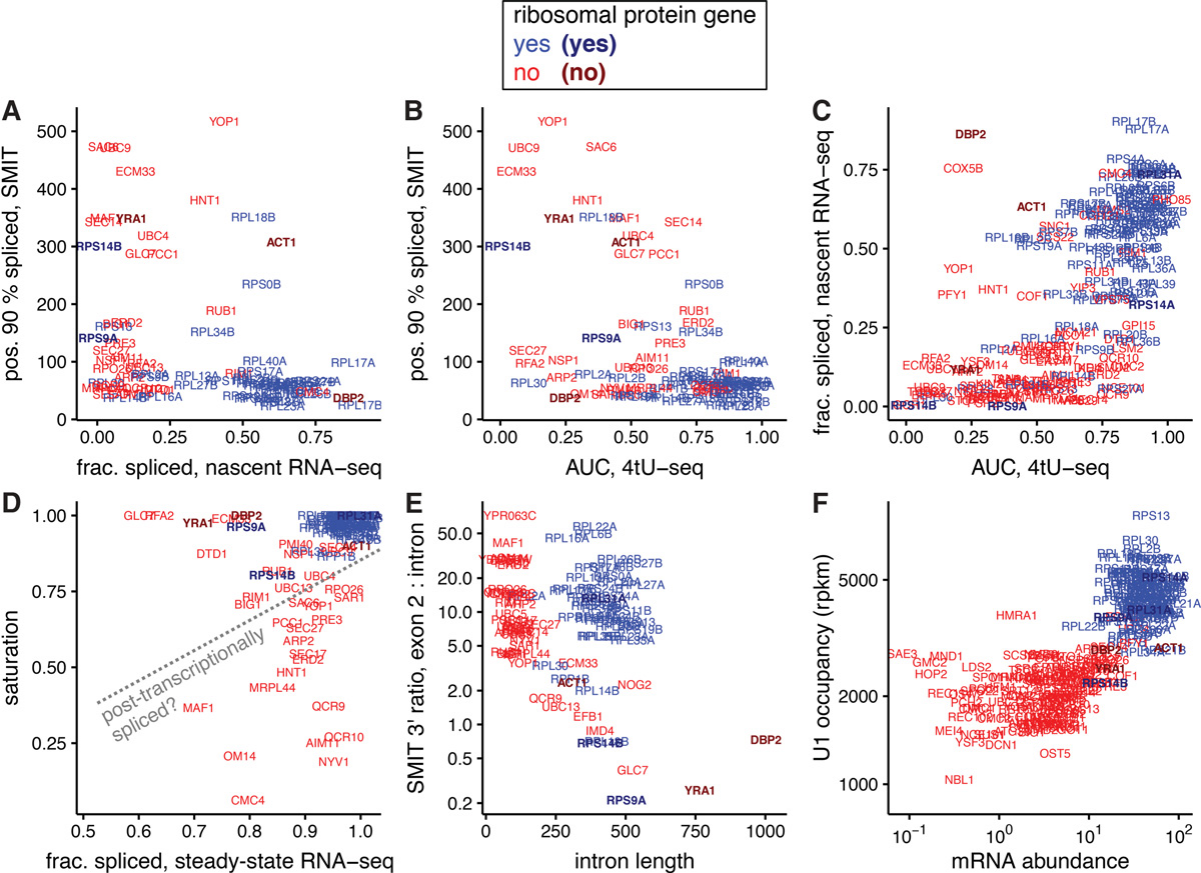
Intron-containing ribosomal protein transcripts (blue) tend to be spliced faster and more cotranscriptionally, compared to nonribosomal transcripts (red). Gene names are plotted with *center* indicating the position on each scale; select genes mentioned in text are shaded and bolded. (*A*) The distance in nucleotides of Pol II from the 3′SS when 90% of transcripts are spliced according to SMIT (pos. 90% spliced) (Carrillo Oesterreich et al. 2016) is plotted versus fraction spliced cotranscriptionally measured by nascent RNA-seq (Harlen et al. 2016). Note pos. 90% was not calculated for *RPL31A* in the SMIT analysis. (*B*) Pos. 90% spliced by SMIT versus AUC by 4tU-seq (a weighted average over the fraction spliced at 1.5, 2.5, and 5 min) (Barrass et al. 2015). (*C*) Fraction spliced according to nascent RNA-seq versus AUC by 4tU-seq. (*D*) Saturation (fraction spliced at most 3′ Pol II positions) (Carrillo Oesterreich et al. 2016) plotted against fraction spliced in steady-state RNA-seq (Barrass et al. 2015). Transcripts of genes *below* the dotted line may be mostly post-transcriptionally spliced. (*E*) Ratio of SMIT 3′ ends in introns and exon 2 (spliced or unspliced) (Carrillo Oesterreich et al. 2016) versus intron length. (*F*) U1 occupancy measured by ChIP-nexus (Harlen et al. 2016) versus mRNA abundance (Csárdi et al. 2015a).

Importantly, SMIT shows that the second step of splicing is complete on most transcripts when Pol II has moved less than 100 nt beyond the 3′SS, consistent with nascent RNA-seq's measure that the majority of splicing is cotranscriptional (Fig. 2A). Also, transcripts that are seen to be spliced fast by 4tU-seq are generally measured as spliced cotranscriptionally by SMIT (Fig. 2B) and nascent RNA-seq (Fig. 2C): in particular, ribosomal protein (RP) transcripts. Transcripts measured by SMIT to be spliced when Pol II is further from the 3′SS appear to be spliced more slowly by 4tU-seq analysis, including the well-characterized *ACT1*. Furthermore, *YRA1*, which is negatively autoregulated by an intron-dependent mechanism (Preker and Guthrie 2006; Dong et al. 2007), is spliced distally, slowly, and inefficiently (Fig. 2A,B). Transcripts that are mostly spliced at steady-state, but have low SMIT saturation values, are candidates for mainly post-transcriptional completion of splicing (Fig. 2D); none of these are RPs.

RP transcripts have other distinguishing patterns in these data: They have relatively fewer SMIT 3′ end reads in the intron compared to exon 2, despite their longer introns (Fig. 2E; Supplemental Fig. S1). This suggests a large decrease in polymerase speed from intron to exon 2, consistent with NET-seq. Furthermore, RP genes have higher U1 occupancy as measured by ChIP-nexus (Harlen et al. 2016), so that the reported “high U1 occupancy genes” are essentially synonymous with the RP genes (Fig. 2F). Notably, the U1 occupancy differs only threefold between RP and non-RP genes, which is much less than the difference in mRNA abundance or, presumably, transcription rate. This is consistent with a low dynamic range or high background signal for U1 occupancy as measured by ChIP, but is also consistent with RP transcripts recruiting more U1 but for shorter times, due to their faster splicing.

These assays reveal differences in splicing between paralogous transcripts, for example, of the *RPS14A* and *RPS14B* genes*.* It has been shown that excess Rps14 protein can bind to a stem–loop structure in *RPS14B* pre-mRNA, inhibiting its splicing and leading to its rapid degradation (Fewell and Woolford 1999). *RPS14B* transcripts are spliced very slowly, inefficiently, and distally compared to other RPs (Fig. 2A–D); *RPS14A* was not measured by SMIT and behaves like most RPs in the other assays (Fig. 2C).

### Some genes are spliced slowly, inefficiently, and/or post-transcriptionally

Curiously, some transcripts are apparently spliced slowly or less efficiently, yet close to the 3′SS: *SEC27, RFA2, NSP1, ARP2, DBP2, OM14, SAR1, RPL2A, RPS9A,* and *RPL30* (lower left quadrant in Fig. 2B). This apparent paradox could reflect the distinction between time and position of splicing: Pol II elongating more slowly or pausing near the 3′SS would allow slow splicing to occur cotranscriptionally. This could be an explanation for proximal splicing of *RPL30* and *RPL2A* transcripts, for which the SMIT saturation value is similar to the fraction spliced at steady state (Fig. 2D). Alternatively, the proximally spliced RNA may represent only a small fraction of the total, with most being spliced post-transcriptionally. For example, *SEC27*, *ARP2*, and *OM14* are spliced inefficiently and proximally, but with low SMIT saturation values (Fig. 2D); in this respect the assays agree. The apparent discrepancy is caused by dividing the low fraction of proximally spliced reads by the still-low fraction of distally spliced reads that indicates mainly post-transcriptional splicing. This conclusion is supported by nascent RNA-seq also measuring a low fraction spliced for *SEC27, RFA2, NSP1, ARP2, OM14, SAR1, RPL2A, RPS9A,* and *RPL30* (lower left quadrant of Fig. 2A).

Artifacts in one or more of the assays could also explain the discrepancy: If an excised intron were degraded particularly slowly and sequenced efficiently, that would depress 4tU-seq estimates of splicing speed. Likewise, SMIT could overestimate the fraction of fully spliced transcripts near the 3′SS if splicing intermediates represent a large fraction of slowly spliced transcripts, as they are not detected in this assay (Box 1).

### Intron-mediated inhibition may be cotranscriptional

The Rpl30 protein negatively regulates splicing of the *RPL30* transcript by binding cotranscriptionally to the intron (Macías et al. 2008). Concordantly, the *RPL30* intron is spliced very slowly, with only 24% spliced after 5 min as measured by 4tU-seq; however, SMIT reports that *RPL30* is 90% spliced when the polymerase has progressed only 70 nt beyond the 3′SS. As suggested above this could be caused by Pol II pausing, with Rpl30-intron binding causing Pol II to pause prior to 3′SS synthesis. The agreement of SMIT saturation with fraction spliced at steady state (Fig. 2D) suggests that, after the hypothesized pause is relieved and Pol II continues past the 3′SS, *RPL30* transcripts are spliced cotranscriptionally. Could other intron-regulated transcripts be regulated by similar cotranscriptional mechanisms?

**FIGURE 3.**
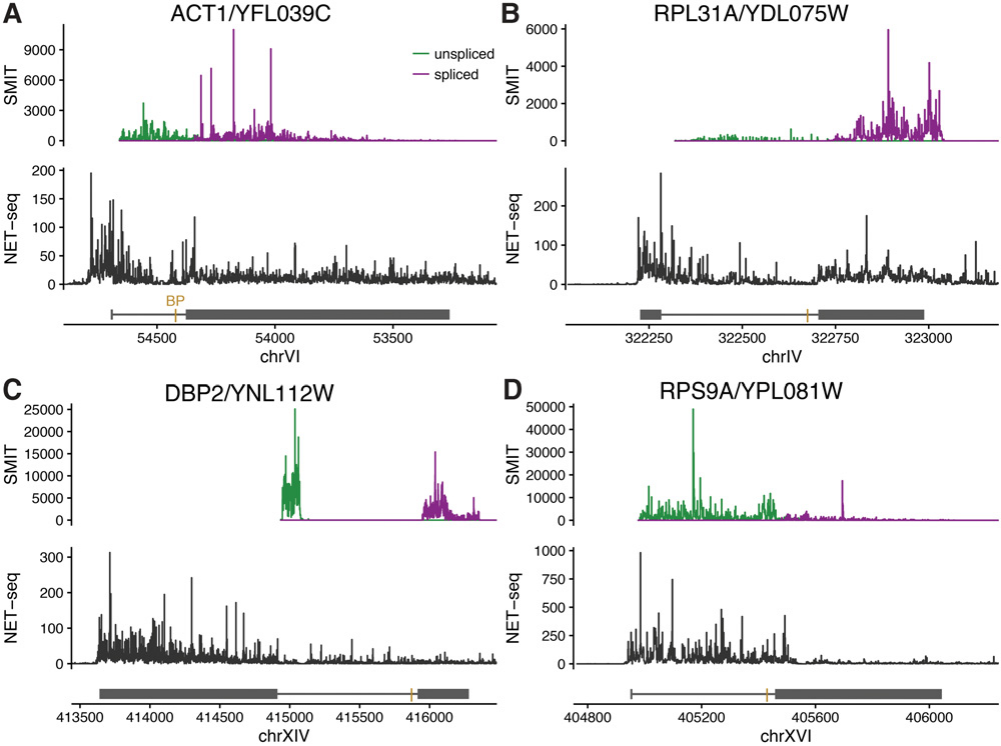
Comparison of SMIT and NET-seq profiles along individual genes, plotted in genomic co-ordinates. *Upper* panels show 3′ end counts of unspliced (green) and spliced (purple) SMIT reads. *Lower* panels show 3′ end counts of NET-seq reads. Line diagrams show exons represented by solid blocks, introns by lines, and the gold bar represents the branch point (BP); these selected genes are all on the plus strand (5′ *left*, 3′ *right*). Note different length and count scales for each gene. (*A*) *ACT1*/YFL039C is spliced with medium efficiency. (*B*) *RPL31A*/YDL075W is a fast-spliced ribosomal protein transcript: The low ratio of intron:exon 2 reads in SMIT, and the depletion of NET-seq reads toward the 3′ end of the intron are typical of RP transcripts. *DBP2*/YNL112W (*C*) and *RPS9A*/YPL081W (*D*) have anomalous profiles.

The Dbp2 protein negatively regulates its own production by binding to its intron to inhibit splicing (Barta and Iggo 1995); its transcript is slowly, yet apparently highly cotranscriptionally, spliced. To investigate at what stage in the transcript's lifetime this regulation might occur, we looked at the SMIT and NET-seq data in more detail (Fig. 3C). The unspliced SMIT reads decline abruptly ∼160 nt into the intron, a position coinciding with a T-rich sequence that is presumably hard to align and also locally absent from NET-seq reads. Thus the apparently complete 3′SS-proximal splicing is due to the near-complete absence of unspliced reads near the 3′SS in the SMIT data. However, NET-seq reads continue along the intron and into exon 2, albeit at a low density compared to exon 1, consistent with incomplete passage of Pol II through the intron. Meanwhile, Dbp2 functions as a cotranscriptional RNA chaperone (Ma et al. 2013, 2016). We hypothesize that the intron-mediated negative autoregulation of *DBP2* splicing occurs cotranscriptionally and results in premature termination or cleavage of the nascent transcript.

Likewise, Rps9b protein binds to the *RPS9A* intron and 3′ UTR in chromatin (suggesting that this happens cotranscriptionally) and represses splicing of the *RPS9A* intron (Petibon et al. 2016). Nevertheless, the *RPS9A* intron is highly cotranscriptionally spliced (according to SMIT) but with low efficiency (according to nascent RNA-seq). Again, both SMIT and NET-seq show a remarkably low density of reads in exon 2 compared to the intron (Fig. 3D). Petibon and colleagues further showed that repression of *RPS9A* splicing is promoter-independent, thus occurs after transcription initiation. We hypothesize that cotranscriptional repression of *RPS9A* splicing by Rps9b protein may result in Pol II pausing or premature termination.

These examples illustrate the rich information that can be obtained by comparing data sets from these different approaches, beyond a simple analysis of splicing efficiency, speed, or position. Indeed, comparing the SMIT profiles on individual genes highlights not only the distinction between RPs and other intron-containing genes, but also the 3′ ends of the intronic snoRNAs embedded in *EFB1* and *IMD4* introns (Supplemental Fig. S1).

More generally, these vignettes emphasize that regulation of transcription is coupled to regulation of splicing.

### Splicing affects transcription elongation and Pol II phosphorylation

Our group and others have observed that elongating Pol II can pause while the nascent pre-mRNA is being spliced. Pol II accumulates transiently, in a splicing-dependent manner, near the 3′ splice site on reporter genes in budding yeast (Alexander et al. 2010), and certain splicing defects give rise to transcription defects at introns, suggesting a transcriptional elongation checkpoint during cotranscriptional spliceosome assembly (Chathoth et al. 2014). Furthermore, Carrillo Oesterreich et al. (2010) reported an apparently distinct transcriptional pausing ∼250 bp downstream from the 3′SS using tiling microarrays of a chromatin fraction. It remains unclear how widespread such pausing phenomena are: At which positions, in which genes, in which organisms, and in which conditions, does transcription wait for splicing, and for how long? Does pausing occur both before and after the first catalytic step of splicing?

Evidence from high-throughput data sets is so far mixed. Carrillo Oesterreich et al. (2016) sequenced 3′ ends of chromatin-associated RNA in a single experiment, reporting no consistent Pol II accumulation close to the 3′SS or at positions of splicing onset or saturation, that might explain 3′SS proximal splicing. The first NET-seq data set (Churchman and Weissman 2011) did not disentangle RNA 3′ ends that could be caused by Pol II pausing from an abundance of reads at the 3′ ends of excised introns. On the other hand, Harlen et al. (2016), with higher read-depth, report an excess of NET-seq reads on yeast exons downstream from the 3′SS relative to upstream in the introns, and a peak immediately downstream from the 3′SS, that is interpreted as Pol II pausing. In agreement, Pol II was seen to accumulate after the 3′SS in a splicing-dependent manner in human cells with NET-seq (Nojima et al. 2015) and with chromatin-derived 3′ end targeted RNA-seq (Mayer et al. 2015).

Splicing also leads to a change in state of the transcription elongation machinery, notably in the modifications of the Pol II CTD. ChIP-qPCR in yeast (Alexander et al. 2010; Chathoth et al. 2014) and ChIP-seq in humans (Nojima et al. 2015) showed a splicing-dependent phosphorylation of Ser5 in the CTD. Furthermore, ChIP-nexus (a modified, more precise, ChIP-seq) (Harlen et al. 2016) reported that Ser5 phosphorylation rises rapidly after the 3′SS in yeast, and Ser5 phosphorylated Pol II is enriched in 5′SS cleaved splicing intermediates in mammalian cells (Nojima et al. 2015). Applying a hidden Markov model to mCRAC data, Milligan et al. (2016) also detected systematic differences between overall modification states of Pol II on intron-containing versus intronless yeast transcripts, with changes of phosphorylation state coinciding with positions of splice sites.

Are these effects of splicing on Pol II functionally related? If specific modifications in Pol II are required for efficient transcription through the second exon, then conditions that alter these modifications could lead to transcriptional pausing. It has been suggested (Alexander et al. 2010; Chathoth et al. 2014) that Pol II pausing and phosphorylation could be evidence for splicing-dependent transcriptional checkpoints, implicating quality control of RNA processing in regulating transcription.

Clearly, more extensive and detailed analyses are needed to characterize RNA processing-dependent polymerase behavior genome-wide. If Pol II pausing happens between the first and second catalytic steps of splicing, detecting the pause via sequencing requires splicing intermediates (specifically, intron–exon lariats) to be quantified simultaneously with the end products. If a subset of (modified) polymerase pauses transiently for splicing, then the signal is likely to be weak in the pool of total polymerase. If polymerase only pauses on a subset of genes, the signal within metagene analyses will be weak. If polymerase pausing is involved in a regulatory response or is modulated by environmental stimuli that affect RNA processing, pausing might be rare in some conditions, but common in others.

### Which features of a transcript determine the cotranscriptionality or speed of splicing?

Intron-containing transcripts differ in the sequences that directly interact with the spliceosome (5′SS, 3′SS, BP) as well as their promoters, untranslated regions, introns, and coding regions, which can affect splicing by forming RNA secondary structure and/or recruiting regulatory proteins. The ideal sequence predictor would explain a substantial proportion of the genewise variance of all splicing measurements consistently, both for RP and non-RP transcripts. Scoring the 5′SS, 3′SS, or BP sequences against their consensus motifs falls far short of this ideal (Supplemental Fig. S2). In contrast, higher intron secondary structure (more negative Δ*G* per nucleotide) correlates with splicing being slower and less cotranscriptional (Supplemental Fig. S2), consistent with the multifactorial analysis in Barrass et al. (2015). However, differences between splicing metrics for RP and non-RP genes are more striking than the differences in these predictors.

### Future directions

Collectively, the data sets examined here provide compelling evidence that RP transcripts are spliced both faster and more cotranscriptionally than the average transcript. RP genes produce the largest and most abundant class of spliced transcripts in yeast (Ares et al. 1999; Cherry et al. 2012), and RP gene expression and splicing are coherently regulated in response to a variety of environmental signals (Pleiss et al. 2007; Bergkessel et al. 2011). The majority of RP introns are required for growth in at least one condition (Parenteau et al. 2011), supporting a regulatory role. RP transcripts are also unusually stable in fast-growth conditions and unusually unstable after glucose starvation (Munchel et al. 2011). Which features of RP genes promote their efficient and cotranscriptional processing? Is it their unusually long (for yeast) introns or their near-consensus GUAUG 5′SS? Is it their stereotypical transcription factors or promoter architecture (Knight et al. 2014), or distinctive complement of interacting RNA-binding proteins (Hogan et al. 2008)? Could it be connected to reciprocal regulation of ribosomal protein and ribosomal RNA production (Lempiäinen and Shore 2009)? Are there distinct spliceosomal factors that favor splicing of RP transcripts? Unraveling the distinctiveness of RP compared to non-RP gene expression is an important ongoing research focus.

The results discussed here were produced using innovative approaches; how robust will the conclusions seem after further methodological development? Protocols, especially those associated with next-generation sequencing, may need extended optimization to distinguish biological sequence-specific signals from noise and artifacts that arise in both sample preparation and data analysis. Ribosome profiling presents an instructive comparison: The initial analysis for yeast reported that ribosomes do not translate rare codons slowly (Ingolia et al. 2009), which was counterintuitive to conclusions from evolutionary biology and biochemistry (Hershberg and Petrov 2008). Active debate continued over several years as many groups adjusted experimental and analytical protocols, particularly regarding the use of ribosome-stalling drugs. Now, there is clear evidence that ribosome profiling does quantify slower translation at rare codons (Hussmann et al. 2015; Weinberg et al. 2016), in agreement with complementary next-generation sequencing approaches (Pelechano et al. 2016).

Nanopore sequencing of nascent RNA would circumvent the length restrictions of short-read Illumina sequencing; direct RNA sequencing promises to circumvent biases caused by reverse-transcription to cDNA (Garalde et al. 2016). Enrichment of spliced transcripts, analogous to SMIT, might be achievable by real-time selective sequencing on a nanopore sequencer (Loose et al. 2016).

The work discussed here has quantified splicing and transcription in the model yeast, *S. cerevisiae,* with unprecedented detail and scope: Multiple experimental approaches applied to the same system yield richer insights than any one viewed alone. Do these observations extend to other eukaryotes? For *Schizosaccharomyces pombe,* fast, cotranscriptional splicing was reported by long-read sequencing (Carrillo Oesterreich et al. 2016) and suggested by 4tU-seq (Eser et al. 2016). NET-seq in mammals supports cotranscriptional splicing, observing Pol II peaks (interpreted as transcriptional pauses) 3′ to a subset of introns (Nojima et al. 2015) and within spliced exons (Mayer et al. 2015). However, very different efficiencies of cotranscriptional splicing have been reported for *Drosophila* and mouse (Khodor et al. 2012). A variety of gene features, including intron, exon and overall gene length, intron position, splice sites and other sequence elements, RNA structure and synthesis rate, contribute to differences in splicing kinetics (Khodor et al. 2012; Barrass et al. 2015; Eser et al. 2016). Explaining such differences is not simple and, clearly, much remains to be learned.

## DATA DEPOSITION

Data were taken from cited publications, where possible with minimal processing, to reflect both the experimental and analysis pipelines used. Nascent RNA-seq data by gene (used in Fig. 1) were a personal communication from K. Harlen, corresponding to Figure 6D of Harlen et al. (2016), and we recomputed the fraction spliced using DICE (Huang and Sanguinetti 2016), after aligning raw nascent RNA-seq reads from GEO (GSE68484; SRR2046809) to the *S. cerevisiae* genome using STAR (Dobin et al. 2013). SMIT data are from Carrillo Oesterreich et al. (2016), Supplemental Table S1, and 4tU-seq data from Barrass et al. (2015), Supplemental Table S7. SMIT intron:exon ratios in Figure 2E were calculated by aligning raw reads from GEO (GSE70908; pooled from smit_20genes and smit_extended data sets, excluding short RNA data) to the *S. cerevisiae* genome using STAR (Dobin et al. 2013); SMIT profiles in Figure 3 and Supplemental Figure S1 were from the same alignments. Intron lengths in Figure 2E were taken from Supplemental Table S8 of Barrass et al. (2015). In Figure 2F, mRNA abundances were taken from Csárdi et al. (2015b) (scer-mrna-protein-absolute-estimate.txt from data package) and U1 occupancy from Supplemental Table S4 of Harlen et al. (2016). NET-seq profiles in Figure 3 are from the bedgraph files (WT_NETseq) in GEO (GSE68484). Data were processed using the statistical language R (R core team) and plotted with ggplot2 (Wickham 2009). R scripts to create the figures are available upon request.

## SUPPLEMENTAL MATERIAL

Supplemental material is available for this article.

## Supplementary Material

Supplemental Material

## NOTE ADDED IN PROOF

As this paper was in press, we were made aware that Schaughency et al. (2014) observed a transcription termination site within the DBP2 intron.
