# Supplementary material for: Extremely fast and incredibly close: cotranscriptional splicing in budding yeast
Source: RNA. 2017 May;23(5):601–10. doi: 10.1261/rna.060830.117 (PMC5393171; doi:10.1261/rna.060830.117)
Supplement: Supplemental Material [file supp_23_5_601__index.html]

Extremely fast and incredibly close: cotranscriptional splicing in budding yeast — Supplemental Material 

# Extremely fast and incredibly close: cotranscriptional splicing in budding yeast

## Supplemental Material

- Supplemental\_FigS1\_SMIT\_nRPvsnonRP\_cumreads.png
- Supplemental\_FigS2\_HarBarrCarr\_vs\_Features.pdf
