# Supplementary figures and images for: Extremely fast and incredibly close: cotranscriptional splicing in budding yeast
Source: RNA. 2017 May;23(5):601–10. doi: 10.1261/rna.060830.117 (PMC5393171; doi:10.1261/rna.060830.117)

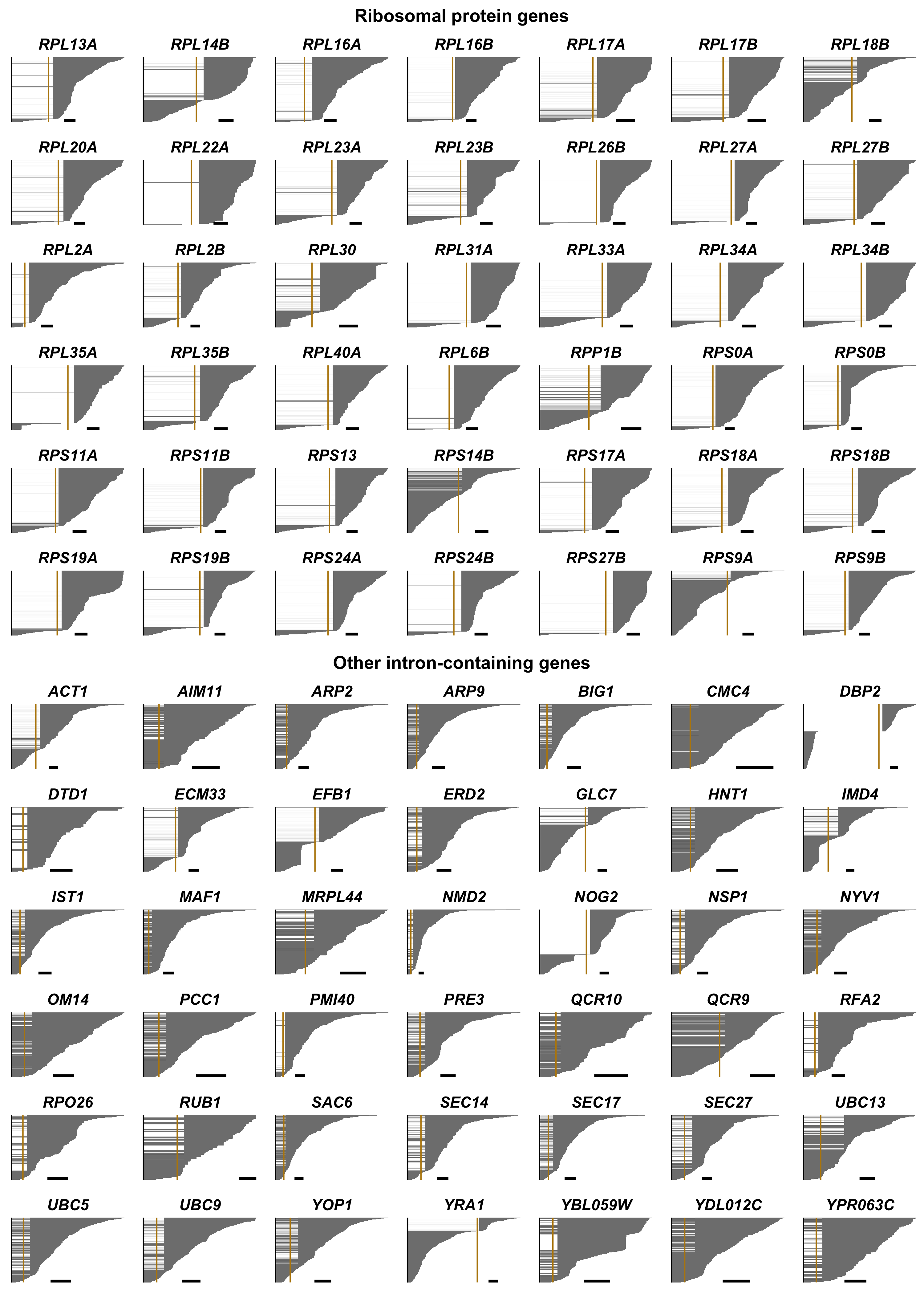

Supplement: Supplemental Material [file supp_060830.117_Supplemental_FigS1_SMIT_nRPvsnonRP_cumreads.png]

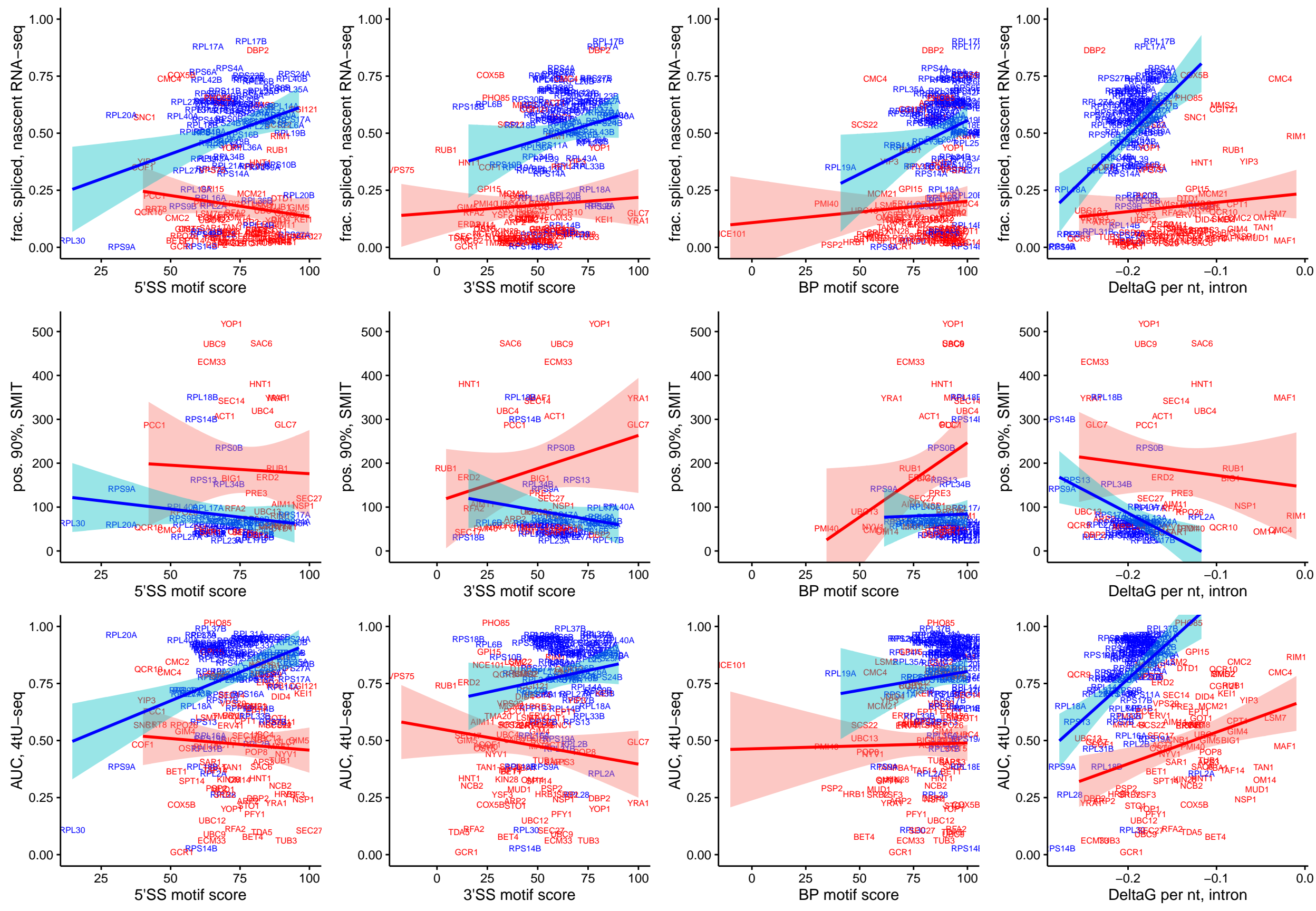

Supplement: Supplemental Material [file supp_060830.117_Supplemental_FigS2_HarBarrCarr_vs_Features.pdf]
